# Supplementary material for: High Temperature Alters Phenology, Seed Development and Yield in Three Rice Varieties
Source: Plants (Basel). 2023 Feb 2;12(3):666. doi: 10.3390/plants12030666 (PMC9921536; doi:10.3390/plants12030666)
Supplement: Supplementary file 1 [file plants-12-00666-s001.zip › plants-2161525-supplementary.pdf]

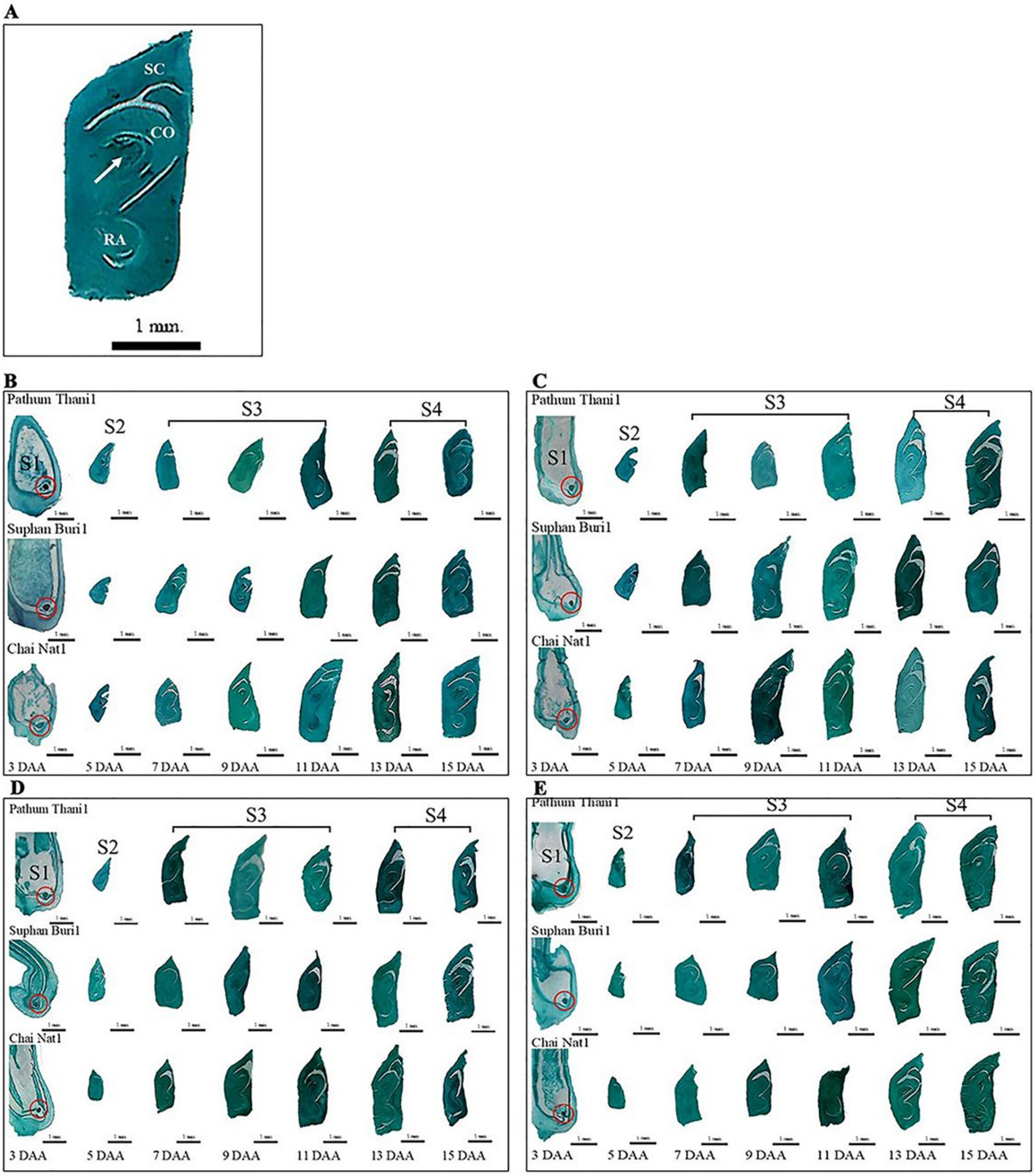

Figure S1. Longitudinal section of changed embryonic development influenced by increased accumulated temperature on rice vars. Pathum Thani 1, Suphan Buri 1 and Chai Nat 1 cultivated on PDI (off-season, B and D) and PDII (late off-season, C and E) in 2018 and 2019. Arrow indicates shoot apical meristem. SC=scutellum, CO=coleoptile, RA=radicle and DAA=days after anthesis. Rice embryonic development shows S1= globular stage (red circle), S2=coleoptile stage, S3=juvenile vegetative stage and S4=maturation stage.

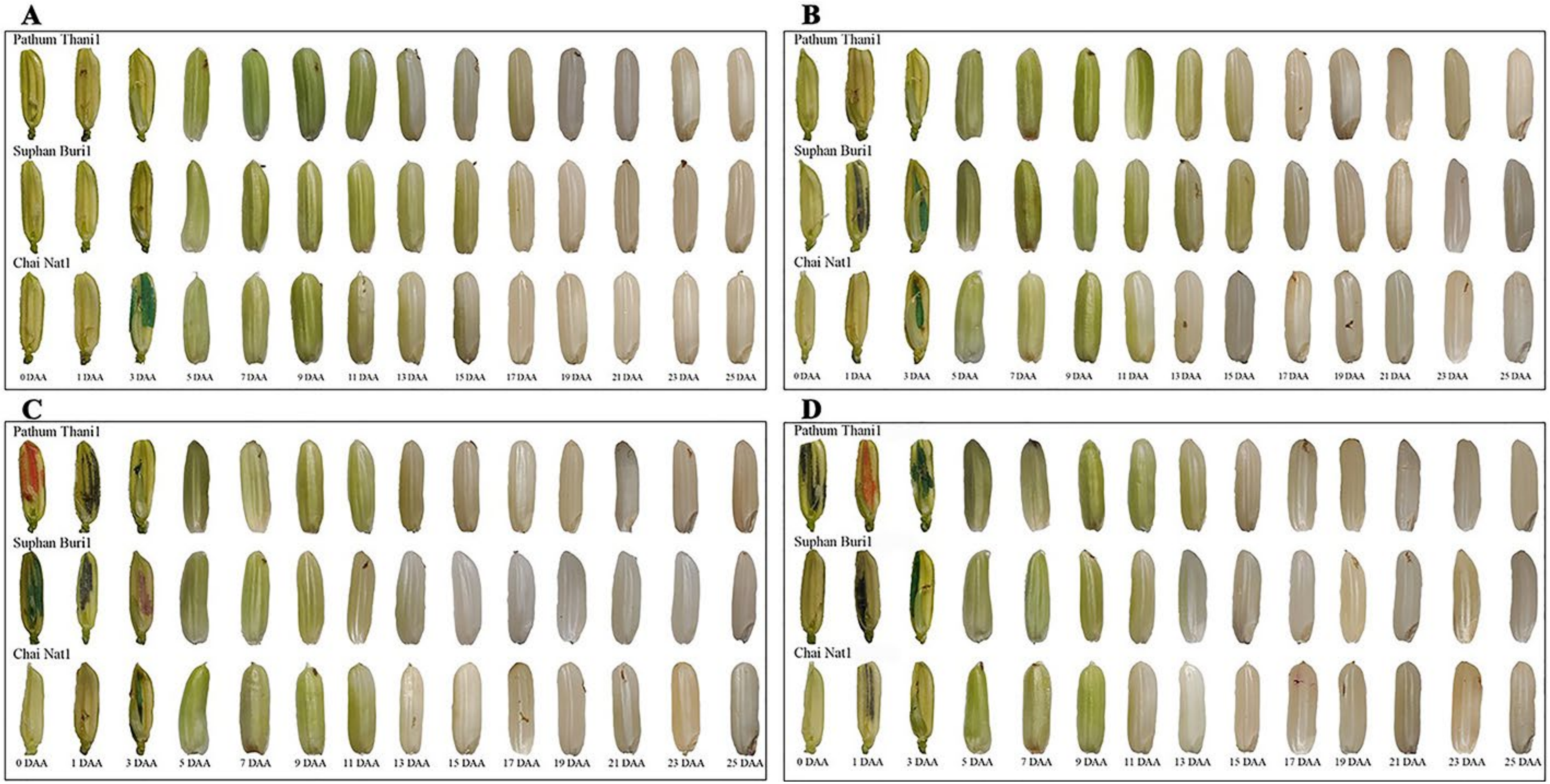

Figure S2. The increased accumulated temperature influenced seed development of rice vars. Pathum Thani1, Suphan Buri1 and Chai Nat 1 cultivated on PDI (off-season, **A** and **C**) and PDII (late off-season, **B** and **D**) in 2018 (**A** and **B**) and in 2019 (**C** and **D**). DAA=days after anthesis.
